# Principles Involved in the Construction of Artificial Teeth

**Published:** 1877-02

**Authors:** C. B. Porter


### PRINCIPLES INVOLVED IN THE CONSTRUCTION OF ARTIFICIAL TEETH.

BY C. B. PORTER, D. D. S.

*Read before the Class in the Dental College of Michigan University.*

One hundred years ago, dentistry, although old as to time, was, as we look back, only in its infancy compared with to-day; the instruments were few, many of them not well adapted for the purposes intended. Then, as now, gold was used to fill decayed teeth, and so were lead and tin. Then, as now, teeth were replanted and transplanted; then, as before, and for some years later, the ivory of the hippopotamus and and elephant were used for the base plate, and teeth for artificial substitutes.

There are many men now living and practicing dentistry, who entered the profession thirty years ago. These men have seen great and rapid changes, almost an entire revolution in the dental art.

See what we have to-day in our operating rooms and laboratories, as compared with thirty years ago, and even to a later day.

The dental chair for the comfort of the patient and the operator, has been the subject of thought and inventive skill of ingenious men. With the present chair, we work with less fatigue and more precision.

Another great improvement is the dental engine, by its use we save much valuable time, besides doing our preparatory work in filling teeth much better, thereby rendering the work more durable, and consequently useful.

The invention and introduction of the rubber dam to the dental profession is of incalculable benefit, an improvement that we could not well do without.

Manufacturers of dental goods are ever ready to furnish the dental depots with all the improved instruments ingenious dentists can invent; thus enabling us, by the use of the best instruments, to make our work on the natural teeth good and durable. Notwithstanding all these improved appliances with the thought, attention and skill, to save the natural teeth, thousands of people, from one cause or another, will lose them, and substitutes must be made.

We have seen rapid advances in operative dentistry, but what can we say of the so-called mechanical department of which I desire more particularly to speak? I fear the tendency exists to an alarming extent among our dental students and with many among those who have recently entered the dental profession to neglect this department of dental science. But I hope to show before I am done that it is a fatal mistake; the need is as great for skilled workmen in the one as the other.

Less than a century ago the teeth of animals and a base-plate of ivory was much used, upon which great skill and ingenious workmanship was bestowed. My own earliest recollection of dentistry was seeing such specimens as these, but bone and ivory gave place to the more enduring metals, gold for the plate with porcelain teeth, which went through various changes; ingenious men adding one improvement after another, till perfection was well nigh attained.

Those skilled in the ceremic art, giving us a beautiful and life-like variety of teeth in size, shape and color, to suit the great variety of cases presented; these teeth so true to nature, with gold for the plate, were, seemingly, all that could be desired for durability and purity, so essential to health and comfort. Then of all the *metals*, gold was king.

When Dr. John Allen brought out his continuous gum work, for full dentures, on platinum for a base plate, and when perfected, and its practicability a settled fact, a long step was taken toward perfection. The excellence of continuous gum work, its life-like appearance in the mouth for dental substitutes, its strength and beauty are such as to entitle it a higher place in the estimation of every dentist who wishes to do the best for his patrons. There is in it adaptability and clealiness, and with due regard to size, shape, color and position, all the essential qualities that go to make up a real substitute for the lost pearls, the beauty of the human face divine, continuous gum work where it can be used, and gold for all others have never been excelled.

But we must not ignore the usefulness of the cast metal plates, rubber and celluloid, because they are cheap, and often short lived materials for dental plates, and because they need but a low order of mechanical skill to work them. They are useful for cheap work, enabling many to wear artificial teeth who could not afford it were precious metals only used. But gold will always stand prominent as the most useful base for artificial teeth in what is called partial plates, and continuous gum probably where it can be used for all others. As this conviction has steadily gained strength with the best talent in our profession, and as time rolls on, has given us years of experience which has enabled us to overcome many difficulties.

It is true the better class of work requires more time, is more difficult to execute, requires more skill to manipulate than any of the other methods now in use. What if it does? If our efforts are rewarded with complete success, why spare the pains? It requires time, perseverance and skill to make any durable and beautiful piece of mechanism; but when it is accomplished, a rich reward awaits him whose efforts have been crowned with success.

So far I have spoken with reference to the best materials for the base plate. Now, let us turn our thoughts for a few moments to a few practical hints in setting the teeth to reach the best results. In setting artificial teeth as they should be, the eye and hand of an artist are requisite to give them color, size and shape, and the highest artistic skill to adjust the substitutes, that they may best subserve the purpose for which they are intended. I have said a dentist must have the eye and hand of an artist to properly select the teeth with referance to size, shape and color, but one thing more is very necessary, *position*. To be well qualified to judge what is required to imitate the natural organs, the dentist must be well trained in the anatomy of the mouth and adjacent parts.

In selecting teeth for artificial dentures, I have found great help in carefully studying the physiognomy and temperament of the patient, in order that the shade and form should harmonize with the rest of the features, which make up the individuality of each particular character, And here let me introduce this axiom which I find in the law of homogeneousness, "Every part of a thing corresponds with every other part and with the whole." Lay before a comparative anatomist a single bone of an unknown animal, he can construct its entire osseous framework.

It is said that Prof. Agassiz was able to do the same thing from a single scale of a fish. The power to do this depends upon a law of comparative anatomy, to which the principle just stated is a counterpart. If it be true, then animal forms are homogeneous. That given a single tooth we can describe every bone in the body, and clothe them in flesh also, if need be. But let us confine our attention now to landmarks posessed by each individual patient seeking our aid and skill. "All parts of the head and face have their fixed relations to each other, and to the character of the person to whom the head and face belongs." There is another member of the body that you should not leave unnoticed in making up your estimate of the need of your patient. The hand, for instance, indicates as much as the face. It reveals its shape and quality also. In fact it is said by physiognomists to be an index of the temperament and make of the whole body. If the hand be long and slender, we find corresponding features and character. Knowing the temperament, and if the complexion be light or dark, you would not be long in selecting a tooth to compare with the individual before you. Also, a plump, round hand goes with a full face, full red lips, a thick nose, and a round head. The oval hand belongs to the oval face, so we might go on showing how every part of a thing corresponds with every other part and with the whole.

Not only should our study extend to the external peculiarities, but to the general temperament and physiological developments as well, and their blendings and modifications.

When the nervous temperament is well marked, all the tissues of the body will be found to be delicate and usually delicate health; smallness of the muscles; features of the face narrow or pointed; fine, thin hair, and a thin delicate skin, and generally a light complexion; rapidity in the muscular actions, vivacity in the sensations. Teeth selected for such a temperament, should be of a light bluish shade, rather slim and narrow, and so arranged as to restore to the mouth its original nervous expression, indicating quickness of motion and active intellectuality. We have another class of people with a well marked phyisognomy. I refer to the vital temperament, and it may be known by a preponderance of the vital or nutritive organs, which occupy the great cavity of the trunk. Its most striking physical characteristic feature is rotundity, the face inclines to roundness; the nostrils are wide; the neck rather short; the shoulders broad and rounded; the chest full; the abdomen well developed; the arms plump and tapering, but terminating in hands relatively small; the complexion generally florid, the countenance smiling, the eyes light, and the hair soft, light and silky. The English furnish some good examples of the vital temperament. When artificial teeth are required for such a person, a very different selection will have to be made from the one first mentioned, in order to meet the requirements of the case. The teeth generally should have a yellowish tint, rather stout and broad, but generally shorter than for one of a nervous temperament, then *position* should be carefully studied to be in keeping with the general make up of the whole body. Another kind of temperament often met with, is what may be called the motive temperament. The figure is commonly tall and striking, if not elegant; the face oblong, the cheek bones rather high, often hard, coarse features, large limbs, dark skin and hair. This temperament is capable of great physical endurance.

Such persons require prominent teeth, dark opaque in color, irregular in position; giving an expression of solidity, firmness and determination of purpose.

These constitute the most striking feature of individuals of marked physiological peculiarity of temperament. It is comparatively an easy task to select and arrange teeth and adapt artificial dentures so as to meet the requirements of those plainly marked cases, but when these various temperaments are more or less blended in one individual, a positive knowledge of physiology and physiognomy is required in order to enable us to blend our work so as to harmonize as a whole. Every part of the face aids in making up individuality, and individuality may be marred or destroyed by a badly selected and improperly arranged set of teeth. The mouth, to say nothing of the words which may issue from it, is the most elegant feature of the face, the center of expression. Every emotion of the mind finds utterance in the silent expression of the lips, revealing to the eye what might never reach the ear, and never find utterance in words, prosperity or adversity, love or hate, mirth or gloom, dignity, firmness, pride, scorn, contempt.

I hope I have suceeded in showing the importance of being thoroughly educated in anatomy and the kindred sciences, so as to successfully restore the irrecoverably lost dentures to their place in the mouth, that they may fulfill all the functions required of them. This I regard as important as it is to treat the natural teeth and diseases of the mouth and adjacent parts.

It will not do to bestow all our energies and knowledge on one department of dentistry and neglect the other. We should never put into less competent hands than our own the direction and exclusive control of the so-called mechanical department of dentistry, and I hope I have succeeded in pointing out the reasons why. One more reason why our mind should direct or work all departments is, the operating dentist has the best opportunities to examine the natural organs *in situ*, to study their *position* and color, the temperament of the individual, color of the hair and skin, thus acquiring a practical knowledge that is indispensable.

But shades and shadows of complexion and expression vary as do the trees of the forest, yet not an individual but has a principle of harmony governing his whole being.

Feb-3

I have endeavored to show you that you will never find light colored hair and eyes with a dark complexion. But the color of the hair, eyes and complexion, the features, in fact the whole physiognomy and formation of the body are all in keeping with the size, shape and color of the teeth. I have spoken emphatically of the temperaments and their influence on the individual, but you will find these temperaments will change with age, and with age also comes a change in the expression of the teeth. Examine carefully the teeth of individuals of twenty to twenty-five and of others of fifty to sixty years of age, and you will see age brings changes in the teeth in perfect keeping with the changes of the features of the face.

When the opportunity occurs, and it often will in your dental practice, to see the best specimens of natural teeth, examine them closely, study them carefully, and you will never find them set in the arch as we too often see artificial teeth, with mathematical exactness, but on the contrary, they always present a graceful irregularity. I would advise every young dentist to note this fact and keep it in mind when arranging the teeth upon a plate. Adhere strictly to nature; follow her and you will not be led astray.

In conclusion let me say one word as to the opportunities you enjoy in this department of Michigan University to acquire a first class dental education. Under the instruction of competent teachers, recommended by their excellent qualifications and experiences, whose hearts are in the work, there is no reason why the students of this school should not take a high and honorable place in the dental profession. Your medical instruction is thorough. Chemistry, a science so essential to the dental student is complete. You have clinical instruction here which can not be excelled. Large numbers of the best people, such as any dentist in the land would be glad to acknowledge as his patients, are here ready to receive from your hands such work as they need. It is to be hoped that you will not fail on your part to improve these splendid opportunities, thus qualifying yourselves to go out into the world to take a high stand in the profession of your choice.

### REPLANTING TEETH.

BY PROF. J. A. WALTING, D. D. S.

The great interest that is just now being manifested, by the dental profession, in the subject of replanting teeth, has induced me to give the history of a few cases, that have come under my observation, and also the conclusions, at which I have arrived regarding the operation, I have had some twenty cases in the last nine months, and I shall refer to such of them as will present the greatest variety of phases. Of these twenty cases nineteen have been successful to one failure. Now under what circumstances are we excusable for resorting to such heroic treatment? It is to meet a certain class of cases, where other treatment is not admissible; as in teeth that have been abscessed for a long time, and do not readily yield to treatment in the mouth, as bicuspids and molars; by this treatment the cause of the disease is removed at once. There is another class of cases where the pulp is largely exposed in very inaccessible cavities, in such we could extract, and remove the pulp, and fill the root or roots in a more perfect manner, than can possibly be done in the mouth.

That this treatment if judiciously followed, will be a great acquisition to dental surgery can not be questioned. That we shall meet with some failures there is no doubt, but the chances are great that good results will greatly predominate and that it must come into quite general practice. I now present a few remarks from my journal.

Case I. Mrs. P., aged thirty-nine; tooth, lower right second bicuspid, posterior approximate cavity, had at some time previously been filled with amalgam. When first seen by me the tooth was very sore and painful; upon removal the root was found very much enlarged by exostosis, which rendered it quite difficult to extract. Upon removing the old filling the pulp was found dead;—the root and cavity were filled with tin foil—the exostosis was filed off, so that it would readily enter the socket in the jaw again, which had been previously plugged with a pledget of cotton, saturated with a weak solution of camphor. The plugging of the socket with some substance that can be readily removed, to prevent the blood from co-agulating, I regard as all important. There was some soreness for three or four days, but at the present time it is just as good and firm as any tooth in her head.

Case No. III. Prof. L; tooth, left superior first bicuspid, small cavity, on the anterior approximate surface. Tooth had been abscessed for a number of years—one cause for the pulp dying. Upon removal the labial root was found to be quite crooked, and around the end for one-eighth of an inch the periosteum was entirely gone, and the root somewhat absorbed—this portion was excised—cavity and roots filled with gold and replaced with but very little pain, little soreness followed. The fistula in the gum closed in a few days and remains healthy and good. I don't think the abscess in this case could have been treated successfully by any other process, as the end of the root was so defective that it was of itself a constant source of irritation. The only remedy was to remove the tooth.

Case No. IV. Mrs. W., aged sixty-one; tooth, lower right cuspid, decay almost encircling the tooth. It had been very sore and painful for two or three months, but had never discharged pus, or had a fistulus opening. Upon removal the root presented a rough and inflammed appearance; the roughness was scraped off, the cavity and root filled with os artificial, it being about the only substance that could be used, the tooth being so frail. It was replaced with some pain and trouble, owing to a neglect to plug the socket, so as to prevent the clot of blood, considerable inflammation and soreness followed for three or four days, when it gradually subsided and the tooth became firm as ever. This case presents more unfavorable conditions than any other that I have treated; the age of the patient, the absorption of the gums, the great amount of inflammation existing in the periosteum, all pointed to a doubtful experiment, but now after six months trial it is a perfect success, being just as firm in its socket as are the other teeth, and it has given no trouble since its recovery.

Case No. V. Mr. H., medical student, age twenty-two; tooth, second lower bicuspid, left side posterior approximate cavity, pulp dead, pus forming at the end of the root. Removed the tooth, filled the crown and pulp cavity with tin foil. The end of the root was shortened a little, tooth replaced without pain, very little soreness followed. The socket in this instance was stopped with a pledget of cotton saturated with Pond's extract of hamamelis, which I have found to work exceedingly well in these cases, as it has a tendency to prevent the blood from co-agulating in the socket, so that upon removal the socket is clean and ready for the immediate replanting of the tooth.

Case No. X. Mr. T. McA., age twenty-five; first lower bicuspid, on left side posterior approximate cavity, pulp dead and pus forming. Tooth was removed, crown and root filled with gold, replaced without pain, socket stopped with chloroform, very little soreness followed, in three days it was so firm that if I had not known it had been replanted I could hardly have persuaded myself to believe it.

In the cases so far enumerated all were with dead pulps, in some fistulous openings had been formed, and of long standing, others just forming pus. Now I will present a few cases, were the pulp was not dead.

Case No. VI. Miss. J., aged seventeen; tooth, first superior bicuspid, left side posterior approximate cavity very large, pulp alive. Tooth was removed, cavity and roots filled with tin foil and replaced without pain; quite sore and on the second day some swelling, which soon subsided. Treated with hamamelis.

Case No. XI. Student; tooth, second lower right bicuspid cavity on posterior approximate surface very large, pulp exposed and partly sloughed off. Tooth was removed and filled with gold, the anterior approximate cavity on the molar adjoining being filled at the same; the tooth was out three hours, but was replaced without difficulty, and to all appearance was to be one of the most satisfactory cases that I have had, but I learned the next day that he had taken a very severe cold, from having a window open before him during the operation, and was confined to his bed, it was a week after the operation that I again saw the tooth. It was free from all soreness, but quite loose, dressed it with tincture of Iodine, directed him to call soon again, but did not see the case again till seven weeks after the operation, when found the tooth was so loose that I removed it with my fingers, it had caused no pain, though there had been great absorption around the root, this is my first and only failure and owing entirely I think to a lack of recuperative power during sickness.

Case No. XII. Mr. L., medical student; tooth, second lower molar, left side, large buccal cavity. Upon removing the tooth the pulp was found to be partially dead or wasted away; filled cavity and roots with tin foil. The roots were straight and went back easily to their places, very little trouble followed and at the present time he is using it just as freely as his other teeth.

Case No. XVII. Miss. B., aged seventeen, second superior bicuspid on right side, posterior approximate cavity, pulp largely exposed and partly wasted away, upon removal the root showed a high state of inflammation, filled it with tin foil. The root was quite straight and the tooth was a little inclined to slip out of the socket, when the mouth was open, the patient was directed to keep the mouth closed for some time. All right in three or four days.

Case No. XX. Mrs., (same patient as number one). Tooth left superior lateral incisor, had a medium sized gold filling which had been in five or six years without trouble, until about a week before I saw the case, at which time the tooth was very sore, pus has commenced to form, pointing to an opening in the roof of the mouth. Upon removal no great amount of foreign matter came away with the root, but marked the place where it had broken off, probing showed a great amount of absorption in the alveolar process. The filling was found to be good, so the pulp cavity was drilled into from the apex of the root and filled with gold, when it was replaced and tied in, to keep it in place, a considerable soreness and pain followed, but subsided in three or four days.

